# In-Depth Analysis Shows Synergy between Erlotinib and miR-34a

**DOI:** 10.1371/journal.pone.0089105

**Published:** 2014-02-14

**Authors:** Jane Zhao, Kevin Kelnar, Andreas G. Bader

**Affiliations:** Mirna Therapeutics, Inc., Austin, Texas, United States of America; Federico II University of Naples, Italy, Italy

## Abstract

Tyrosine kinase inhibitors directed against epidermal growth factor receptor (EGFR-TKI), such as erlotinib, are effective in a limited fraction of non-small cell lung cancer (NSCLC). However, the majority of NSCLC and other cancer types remain resistant. Therapeutic miRNA mimics modeled after endogenous tumor suppressor miRNAs inhibit tumor growth by repressing multiple oncogenes at once and, therefore, may be used to augment drug sensitivity. Here, we investigated the relationship of miR-34a and erlotinib and determined the therapeutic activity of the combination in NSCLC cells with primary and acquired erlotinib resistance. The drug combination was also tested in a panel of hepatocellular carcinoma cells (HCC), a cancer type known to be refractory to erlotinib. Using multiple analytical approaches, drug-induced inhibition of cancer cell proliferation was determined to reveal additive, antagonistic or synergistic effects. Our data show a strong synergistic interaction between erlotinib and miR-34a mimics in all cancer cells tested. Synergy was observed across a range of different dose levels and drug ratios, reducing IC_50_ dose requirements for erlotinib and miR-34a by up to 46-fold and 13-fold, respectively. Maximal synergy was detected at dosages that provide a high level of cancer cell inhibition beyond the one that is induced by the single agents alone and, thus, is of clinical relevance. The data suggest that a majority of NSCLC and other cancers previously not suited for erlotinib may prove sensitive to the drug when used in combination with a miR-34a-based therapy.

## Introduction

Lung cancer accounts for the most cancer-related deaths in both men and women [1]. Targeted therapies are used depending on the cancer genotype or stage of disease and includes erlotinib, a small molecule inhibitor directed against epidermal growth factor receptor (EGFR). Erlotinib functions as competitive inhibitor of ATP-binding at the active site of the EGFR kinase [2]. Clinical trials investigating EGFR inhibitors revealed that responses occurred in a selective fraction of lung cancer patients, preferentially in never-smokers diagnosed with activating mutations in the *EGFR* gene and a adenocarcinoma or bronchioalveolar histotype [3], [4]. However, a majority of non-small cell lung cancer (NSCLC) patients remained resistant. Primary and secondary resistance has been associated with activating *KRAS* mutations that may co-exist with *EGFR* mutations despite the fact that *KRAS* and *EGFR* mutations appeared to be predominantly mutually exclusive [5], [6], an acquisition of a second mutation in the catalytic domain of EGFR (usually T790M) [7], an amplification and overexpression of receptor kinase *MET* and its ligand *HGF*, providing signals into the PI3K pathway and substituting for an inactivation of EGFR [8], increased expression of the receptor kinase *AXL* and its ligand *GAS6* [9], [10], and several others [11]–[15].

The therapeutic use of tumor suppressor miRNAs continues to raise much attention because they can interfere with many different oncogenic pathways at once by broadly targeting multiple oncogenes [16], [17]. Certain miRNAs can also suppress the properties of cancer stem cells, a cell species particularly resistant to many several cancer therapies [18], [19]. Because of these features, synthetic mimics of tumor suppressor miRNAs have become attractive tools to battle cancer as mono-therapies but also to break resistance mechanisms, making current treatment regimens more effective. An example is provided by miR-34a that inhibits a broad range of cancer cell types in culture and in preclinical animal studies [20]. A liposomal formulation, MRX34, loaded with miR-34a mimics has recently entered clinical testing for patients with hepatocellular carcinoma (HCC) and other cancers with metastatic lesions in the liver, including cancers of the lung [21]. There are several studies demonstrating how miR-34a can sensitize cancer cells to conventional cytotoxic chemotherapies [18], [22]–[26]. However, the combination of tumor suppressor miRNAs with targeted therapies is a less well studied approach. Due to the broad anti-oncogenic activity of miR-34a, and the fact that both *MET* and *AXL* are directly repressed by miR-34a [27]–[29], we rationalized that miR-34a can sensitize cancer cells to erlotinib.

Here, we employed multiple analytical approaches to distinguish between additive, antagonistic and synergistic drug interactions and studied the effects of the erlotinib-miR-34a combination in a panel of NSCLC and HCC cell lines with primary or acquired erlotinib resistance. Our data indicate strong synergy in all cell lines tested for various drug-drug ratios. Importantly, miR-34a and erlotinib cooperated synergistically at dose levels that induce maximal cancer cell inhibition, one that is greater than the inhibition achieved by either agent alone. Thus, our results demonstrate how the therapeutic application of erlotinib can be expanded to other cancers and point to a novel combination therapy that could quickly be implemented in the clinic.

## Materials and Methods

### Cell Lines

Human non-small cell lung cancer (NSCLC) cell lines A549, H460, H1299, H226, HCC827 parental and HCC827^res^ were used to assess the combinatorial effects of miR-34a and EGFR-TKIs. The particular cell lines were selected based on the high IC_50_ values of EGFR-TKIs in these cells, their oncogenic properties and susceptibility to miRNAs. These cell lines are either erlotinib resistant (A549, H460, H1299, H226) or sensitive (HCC827). In addition, cell lines with acquired resistance were created by applying increased selective pressure of erlotinib over ten weeks, starting at an equivalent of IC_10_ and ending at an IC_90_ equivalent. As cellular proliferation exhibited normal doubling rates under IC_90_ selection, the resistant cells were plated at a low dilution (HCC827^res^) or high dilution to create near-pure, erlotinib resistant clones (HCC827^res^-#5,6 and 7). To study effects in hepatocellular carcinoma (HCC) cells, Hep3B, Huh7, C3A and HepG2 were used. Huh7 cells were acquired from the Japanese Collection of Research Bioresources Cell Bank. All other parental cells were purchased from the American Type Culture Collection (ATCC, Manassas, VA) and cultured according to the supplier’s instructions.

### RNA Isolation and qRT-PCR

Total RNA from cell pellets was isolated using the mirVANA PARIS RNA isolation kit (Ambion, Austin, TX) following the manufacturer’s instructions. RNA concentration was determined by absorbance measurement (A260) on a Nanodrop ND-1000 (Thermo Scientific, Wilmington, DE). For the quantification of miRNA and mRNA by quantitative reverse-transcription polymerase chain reaction (qRT-PCR), we used commercially available reagents. The RNA was converted to cDNA using MMLV-RT (Invitrogen, Carlsbad, CA) under the following conditions: 4°C for 15 min; 16°C for 30 min; 42°C for 30 min; 85°C for 5 min. Following cDNA synthesis, qPCR was performed on 2 µL of cDNA on the ABI Prism 7900HT SDS (Applied Biosystems, Life Technologies, Foster City, CA) using Platinum Taq Polymerase (Invitrogen) under the following cycling conditions: 95°C for 1 min (initial denature); then 50 cycles of 95°C for 5 sec, 60°C for 30 sec. TaqMan Gene Expression Assays and TaqMan MicroRNA Assays were used for expression analysis of mRNA and miRNA in all lung and liver cell lines. For miRNA expression, additions to the manufacturers’ reagents include DMSO (final concentration of 6%) and tetramethylammoniumchloride (TMAC; final concentration of 50 mM in both RT and PCR) to improve the slope, linearity and sensitivity of the miRNA assays. Expression levels of both miRNA and mRNA were determined by relative quantitation to the HCC827 parental cell line. The raw Ct values of the miRNA and mRNA targets were normalized to selected housekeeping genes to create delta-Ct values, converted to linear space and then expressed as percentage expression.

### miRNA and Erlotinib Treatment

Erlotinib hydrochloride was purchased from LC Laboratories (Woburn, MA). Synthetic miR-34a and miR-NC mimics were manufactured by Life Technologies (Ambion, Austin, TX). To determine the IC_50_ value of each drug alone, 2,000–3,000 cells per well were seeded in a 96-well plate format and treated with either erlotinib or miR-34a as follows. (i) miR-34a mimics were reverse-transfected in triplicates in a serial dilution (0.03–30 nM) using RNAiMax lipofectamine from Invitrogen according to a published protocol [30]. As controls, cells were also transfected with RNAiMax alone (mock) or in complex with a negative control miRNA mimic (miR-NC). Cells were incubated with AlamarBlue (Invitrogen) 4 days or 6 days post transfection to determine cellular proliferation of lung or liver cancer cells, respectively. Proliferation data were normalized to mock-transfected cells. (ii) Erlotinib, prepared as a 10 and 20 mM stock solution in dimethyl sulfoxide (DMSO), was added to cells one day after seeding at a final concentration ranging from 0.1 and 100 µM. Solvent alone (0.5% final DMSO in H226 and HCC827, 1% final DMSO in all other cell lines) was added to cells in separate wells as a control. Three days thereafter, cellular proliferation was measured by AlamarBlue and normalized to the solvent control.

### Regression Trendlines & IC_50_ Values

Linear and non-linear regression trendlines were generated using the CompuSyn (ComboSyn, Inc, Paramus, NJ) and Graphpad (Prism) softwares, respectively. The non-linear trendlines provided a better fit for the actual data and were used to calculate IC_50_, IC_25_ and other drug concentrations (IC_x_), although both softwares generated similar values.

### Combination Effects Determined by the “Fixed Concentration” Method

The “Fixed Concentration” method was used for cell lines with acquired resistance (HCC827^res^). Cells were reverse-transfected with miR-34a using the miRNA at a fixed, weak concentration (∼IC_25_) as described above. The following day, cells were treated with erlotinib in a serial dilution (0.01–100 µM). Cell proliferation inhibition was analyzed 3 days later by AlamarBlue. To measure the effects of the single agents and to correct for effects potentially contributed by lipid carrier or vehicle, cells were also treated with miR-34a in combination with solvent (0.5% DMSO in HCC827^res^, 1% DMSO in all other cell lines) or erlotinib in combination with mock-transfection. All proliferation data was normalized to mock-transfected cells treated with solvent (DMSO). The combinatorial effect was evaluated by a visual inspection of the erlotinib dose-response curve and a shift of the IC_50_ value in the presence or absence of miR-34a (graphed and calculated using Graphpad).

### Combination Effects Determined by the “Fixed Ratio” Method

Cells were treated with 7 concentrations of erlotinib each in combination with 7 concentrations of miR-34a. Each drug was used at a concentration approximately equal to its IC_50_ and at concentrations within 2.5-fold (NSCLC) or 2-fold (HCC) increments above or below. This matrix yielded a total of 49 different combinations representing 13 different ratios. Each drug was also used alone at these concentrations. miR-34a and erlotinib were added as described above, and cellular proliferation was determined by AlamarBlue. Each data point was performed in triplicates.

### Calculation of Combination Index (CI) Values

Combination index (CI) values based on Loewe’s additivity model were determined to assess the nature of drug-drug interactions that can be additive (CI = 1), antagonistic (CI>1), or synergistic (CI<1) for various drug-drug concentrations and effect levels (Fa, fraction affected; inhibition of cancer cell proliferation) [31]–[33]. Both linear regression and nonlinear regression trendlines were used to calculate and compare CI values. CI values based on linear regression analysis was done using the CompuSyn software (ComboSyn Inc., Paramus, NJ), following the method by Chou et al., whereby the hyperbolic and sigmoidal dose-effect curves are transformed into a linear form [31], [34]. CI values derived from non-linear regression trendlines were calculated using **Equation 1** in which C_A,x_ and C_B,x_ are the concentrations of drug A and drug B in the combination to produce effect X (Fa). IC_x,A_ and IC_x,B_ are the concentrations of drug A and drug B used as a single agent to produce that same effect.
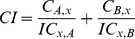
(1)


Drug concentrations required in Equation 1 to determine CI values (C_A,x_, C_B,x_, IC_x,A_ and IC_x,B_) were calculated using the Hill equation (**Equation 2**), IC_50_ and Hill slope value (n) derived from non-linear regression trendlines (Graphpad).
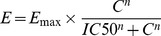
(2)


### Isobolograms

To describe the dose-dependent interaction of erlotinib and miR-34a, isobolograms at effect levels of 50% and 80% inhibition of cancer cell proliferation were created. Since the single agents – alone or in combination – usually reached 50% cancer cell inhibition, the 50% isobologram provided an actual comparison of the single use vs. the combination. The 80% isobologram was used to illustrate the utility of the combination at a high effect level that have practical implications in oncology. In each of these, additivity was determined by extrapolating the dose requirements for each drug in combination from its single use (IC_50_, IC_80_). Data points above or below the line of additivity indicate antagonism or synergy, respectively. For all 49 combinations, drug concentrations required in the combination were compared to those of the single agents alone to reach the same effect and expressed as a fold change (dose reduction index, DRI).

### Curve Shift Analysis

To allow a direct comparison of the dose-response curves and to identify synergistic drug-drug interaction, non-linear regression trendlines of each drug alone or of the combination (IC_50_:IC_50_ ratio or other ratios where indicated) were normalized to its own IC_50_ value and referred to as IC_50_ equivalents (IC_50_ eq). IC_50_ equivalents of the combination were calculated using **Equation 3** and described in [35]. Data of the single agents and in combination were graphed in the same diagram to illustrate lower drug concentrations required to achieve any given effect relative to the single agents. This is represented in a left-shift of the dose-response curve and indicates synergy [35].
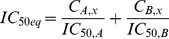
(3)


### Statistical Analysis

Statistical analysis was done using the Excel (Microsoft), CompuSyn and Graphpad softwares. Averages and standard deviations were calculated from triplicate experiments. Goodness of fit of linear and non-linear regression trendlines was described by R (CompuSyn) and R^2^ (Graphpad) values, respectively, and were >0.9 for most cell lines except H226 and HepG2 cells due to limiting drug insensitivity.

## Results

### miR-34a Restores Sensitivity to Erlotinib in Non-small Cell Lung Cancer Cells

**Figure 1:**
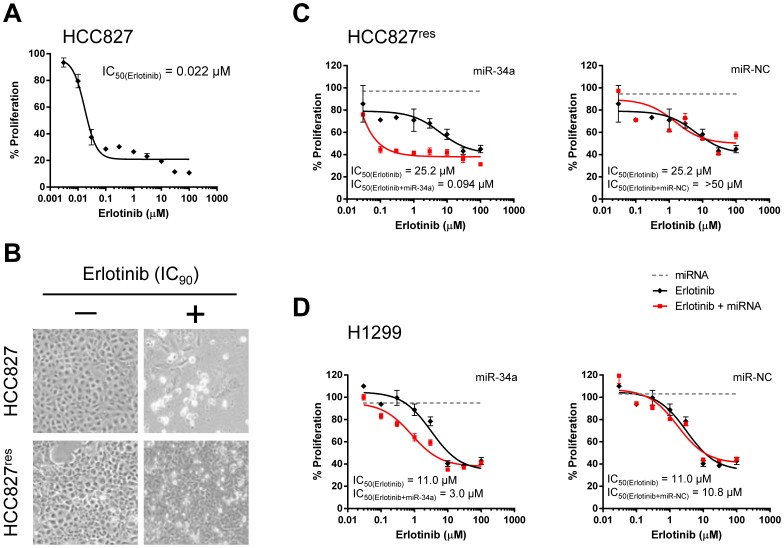
miR-34a restores sensitivity to erlotinib in non-small cell lung cancer cells. (**A**) Dose-dependent effect of erlotinib in parental HCC827 cells. Cells were treated with erlotinib in a serial dilution for 3 days, and cellular proliferation was determined by AlarmaBlue. (**B**) HCC827 cells resistant to erlotinib (HCC827^res^) were developed by incubating cells with increasing erlotinib concentrations over the course of 10 weeks until cells grew normally at concentrations equal to IC_90_ in parental HCC827. (**C, D**) HCC827^res^ and H1299 cells were reverse-transfected with 0.3 nM miR-34a or miR-NC, and incubated in media supplemented with erlotinib in a serial dilution. After 3 days, cellular proliferation was determined. IC_50_ values of erlotinib alone or in combination with miRNA are shown in the graphs.

To study drug resistance in cells with acquired resistance, we used HCC827 cells that express an activating EGFR mutation (deletion of exon 19 resulting in deletion of amino acids 746–750). HCC827 are highly sensitive to erlotinib with an IC_50_ value of 0.022 µM (**Fig. 1A**). Erlotinib-resistant cell lines were developed by exposing the parental HCC827 cells to increasing erlotinib concentrations over the course of 10 weeks until the culture showed no signs of growth inhibition at a concentration that is equivalent to IC_90_ in the parental cell line (**Fig. 1B**). During this process, individual cell clones (HCC827^res^-#5, #6, #7) as well as a pool of resistant cells (HCC827^res^) were propagated. Total RNA was isolated and probed by quantitative PCR for levels of miR-34 family members and genes known to induce resistance. In agreement with previously published data, HCC827 cells resistant to erlotinib showed increased mRNA levels of MET and its ligand HGF that presumably function to bypass EGFR signaling (**Fig. S1**) [8]. In contrast, expression levels of other genes also associated with resistance, such as *AXL*, *GAS6*, *KRAS*, *FGFR1*, *ERBB3*, *PIK3CA* and *EGFR* itself, were not elevated. Levels of miR-34b/c family members were reduced in several of the resistant HCC827 cells (**Fig. S1**). Interestingly, miR-34a was not reduced in erlotinib-resistant HCC827 cells suggesting that miR-34a does not play a causal role in the onset of resistance in these cells which can occur independently of miR-34 by amplification of the *MET* gene [8].

Since both *MET* and *AXL* are directly repressed by miR-34, and because inhibition of AXL can antagonize erlotinib resistance [10], [36], the introduction of synthetic miR-34 mimics may restore erlotinib sensitivity. To explore this possibility, HCC827^res^ cells were exposed to increasing erlotinib concentrations, ranging from 0.03–100 µM, either in the absence or presence of miR-34a used at a fixed, weak concentration (0.3 nM). The effects of erlotinib were expected to be concentration-dependent, such that erlotinib in combination with miR-34a produced lower IC_50_ values relative to erlotinib alone. As shown in **Figure 1C**, erlotinib was not very potent in HCC827^res^ cells (IC_50_ = 25.2 µM). However, when used in combination with miR-34a, the erlotinib IC_50_ value decreased to 0.094 µM. This result suggests that adding a small amount of miR-34a is capable of restoring erlotinib sensitivity that is similar to the one of parental HCC827 cells. The effects were specific to the miR-34a sequence as the addition of a negative control miRNA (miR-NC) did not improve the potency of erlotinib (**Fig. 1C**). Thus, the data generated in HCC827^res^ cells indicate that miR-34a can sensitize cancer cells with acquired erlotinib resistance.

To determine whether the miRNA can also counteract primary resistance mechanisms, we used H1299 cells that have mutations in the *NRAS* and *TP53* genes [37]. In these cells, erlotinib produced an IC_50_ value of 11.0 µM (**Fig. 1D**) in accord with previous results showing EGFR-TKI IC_50_ values between 8.6 and 38 µM [38], [39]. In combination with 0.3 nM miR-34a, the erlotinib dose-response curve shifted along the x-axis, indicating an approximately 4-fold lower IC_50_ value (3.0 µM). This result is in contrast to miR-NC that did not alter the potency of erlotinib, and suggests that miR-34a sensitizes non-small lung cancer cells with both acquired as well as primary resistance.

### miR-34a and Erlotinib Synergize in Non-small Cell Lung Cancer Cells

**Figure 2:**
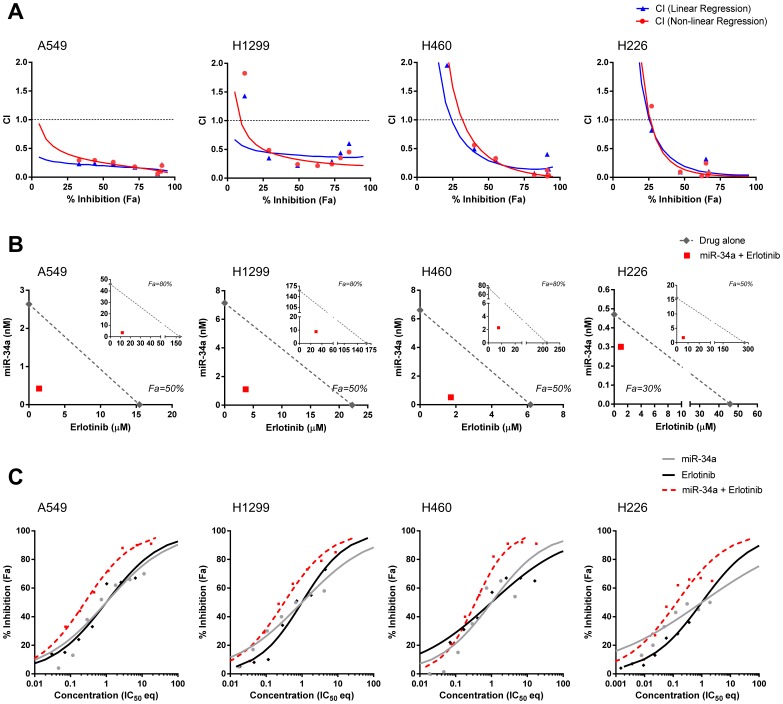
Synergistic effects between miR-34a and erlotinib in NSCLC cells. (**A**) Combination index (CI) analysis. CI values were generated by linear regression (red) and non-linear regression methods (blue). Trendlines indicate CI values at any given effect (Fa, fraction affected, % inhibition), and symbols represent CI values derived from actual data points. CI = 1, additivity; CI>1, antagonism; CI<1, synergy. (**B**) Isobologram analysis. The diagonal, dotted line indicates additivity, and the red symbol shows dose requirements to achieve 50% and 80% (A549, H1299, H460) or 30% and 50% (H226) cancer cell inhibition, respectively. Data points below the line of additivity indicate synergy, data points above denote antagonism. (**C**) Curve shift analysis. Data derived from non-linear regression trendlines were normalized to IC_50_ values of the single agents (IC_50_ eq) and plotted in the same graph. Left and right shifts of the dose-response curves of the combination (red dotted line) relative to the dose-response curves of the single agents (grey, black) indicate synergy or antagonism, respectively. Actual experimental data points are shown (symbols).

The shift of the erlotinib IC_50_ value demonstrated how a fixed miR-34a concentration can improve the potency of erlotinib. However, this model, also known as “Fixed-Concentration-Model”, does not allow the assessment of synergy. To investigate whether both drugs can enhance each other, we employed the “Fixed-Ratio-Model” that is based on Loewe’s concept of additivity [31]–[33]. In this model, combination index (CI) values are calculated based on the slope and IC_50_ value of each dose-response curve (drug alone or in combination) and define whether the drug-drug interactions are synergistic (CI<1), additive (CI = 1), or antagonistic (CI>1). Since the accuracy of the CI values depends on the fit of the dose-response curve trendline, CI values were calculated by two methods using either linear or non-linear regression trendlines (see Materials and Methods). Four erlotinib-resistant cell lines were used, all of which differ in their genetic make-up: A549 (mutations in *KRAS, STK11, CDKN2A*), H460 (mutations in *KRAS, STK11, CDKN2A, PIK3CA*), H1299 (mutations in *NRAS, TP53*), and H226 (mutations in *CDKN2A*) [37]. A qRT-PCR analysis showed a marked increase of *AXL*, *GAS6* and *FGFR1* mRNA levels in these cells relative to erlotinib-sensitive HCC827 cells, further providing an explanation for erlotinib resistance (**Fig. S1**). Levels of miR-34 were significantly reduced in H1299 and H460 cells. In a first step, erlotinib or miR-34a were added to cells in a serial dilution to determine IC_50_ values of each drug alone. For erlotinib, these ranged between 4.2 and >50 µM and are within the range of published data [38]–[42] (**Fig. S2**). The IC_50_ values of miR-34a ranged from 0.4 to 15.6 nM. Neither drug was capable of 100% cancer cell inhibition, nor did the maximal activity of either drug exceed 75%. Erlotinib and miR-34a were least effective in H226 cells, yielding theoretical IC_50_ values as a result of an extrapolation of the dose-response curve. In a second step, each drug was combined at a concentration equal to its own approximate IC_50_ value, as well as at multiples thereof above and below (fixed ratio). As controls, each drug was used at these concentrations alone. Both linear and non-linear regression models produced CI values that are well below 1.0 in all cell lines tested indicating strong synergy (**Fig. 2A**). CI values we considered relevant are those below 0.6. In most cell lines, synergy was observed at higher dose levels and at higher magnitude of cancer cell inhibition. This is critical because a practical application of the drug combination calls for synergy at maximal cancer cell inhibition (75% inhibition or greater). In general, the non-linear regression trendline provided a better fit for the actual data, although both models generated similar results.

Next, we generated isobolograms and determined the dose requirements for each drug at 50% and 80% cancer cell inhibition as a read-out for synergy. The 50% effect level was chosen because the potency of a drug is frequently assessed at its IC_50_ and because in our studies each drug alone was capable of inhibiting most cancer cells by 50%, allowing a comparison of each drug alone with the combination within the range of actual data. The 80% effect level was chosen because it is important to demonstrate synergy at high inhibitory activity for oncology applications. Although the concentrations of each drug alone to achieve 80% inhibition are based on an extrapolation of the dose-response curve and are theoretical in nature, the miR-34a-erlotinib combination readily achieved 80% inhibition or greater and is within the range of actual data. Since the two drugs by themselves were not very effective in H226 cells, isobolograms at 30% and 50% inhibition were created for H226 data. As shown in **Figure 2B**, the isobole of the combination was well below the additive isobole for every cell line and effect level indicating strong synergy. The dose requirement for erlotinib decreased to 2 µM or less in most cell lines to achieve 50% inhibition, reducing the dose by 4- to 46-fold. Likewise, the required concentration of miR-34a was also substantially less in the combination relative to miR-34a alone, reducing its dose by 7- to 13-fold. This reduction in dose level, also referred to as dose reduction index (DRI), was markedly evident at 80% inhibition at which the dose requirements were reduced by up to 28-fold (erlotinib) and 33-fold (miR-34a).

Third, we performed curve-shift analyses whereby the concentration of each drug has been normalized to its own IC_50_ value [35]. This conversion of drug concentrations into IC_50_ equivalents (IC_50_ eq) allows a direct comparison of each dose-response curve from the single agents and the combination. Trendlines were generated and span effect levels from 0–100% inhibition. The slope of the trendline indicates drug potency, and the maximal activity can be gaged from actual data points. Synergy is identified when IC_50_ equivalents of the combination are lower to achieve any given effect relative to the single agents [35]. This is visually indicated by a left-shift of the combination trendline. As seen in **Figure 2C**, the combination is well separated from the single agents indicating synergy. In H460 and H226 cells, the IC_50_ equivalents of the combination are greater at low effect levels (0–25%) and lower at effect levels above 30% compared to those of the single agents. This observation agrees with data from CI plots showing antagonism below 25% inhibition and synergy above 25% inhibition in these cells (**Fig. 1A**). Thus, the analysis reveals synergistic effects for drug concentrations that induce a high level of cancer cell inhibition. A benefit for the combination is further demonstrated by the fact that the actual level of inhibition is greater for the combination relative to the single agents – the maximal activity of the single drugs is no greater than 75% and can be extended beyond 90% when used in combination.

### Multiple Ratios of Erlotinib and miR-34a Cooperate Synergistically

**Figure 3:**
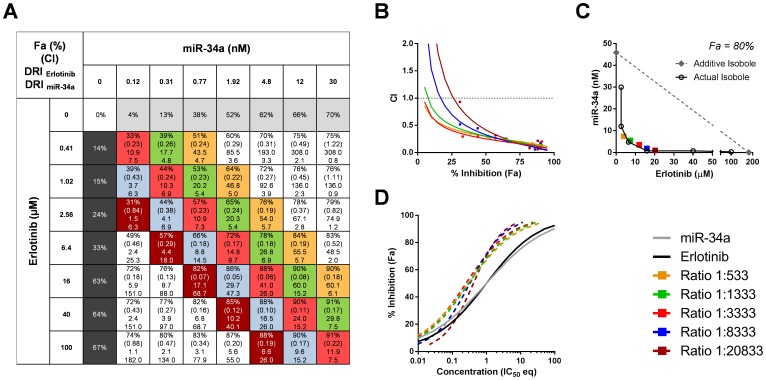
Multiple ratios of erlotinib and miR-34a cooperate synergistically in A549 cells. (**A**) Summary table showing potency (Fa), CI and DRI values of erlotinib and miR-34a combined at various concentrations and ratios. The molar miR-34-erlotinib ratios 1∶533, 1∶1333, 1∶3333 (IC_50_:IC_50_ ratio), 1∶8333, and 1∶20833 are indicated in yellow, green, red (IC_50_:IC_50_ ratio), blue and maroon. (**B**) Combination index plot of various drug ratios. CI values from actual data points are indicated by symbols. (**C**) Isobologram at 80% cancer cell inhibition. Colored symbols represent the 80% isobole of various ratios. The black line represents the isobole derived from actual erlotinib-miR-34a combinations that produced 80% (±2%) inhibition. (**D**) Curve shift analysis of various drug ratios.

Our analysis suggests that erlotinib and miR-34a synergize when the two drugs are combined at a ratio derived from their IC_50_ values. Because drug-drug interactions can change depending on the relative amounts, we explored the effects of multiple erlotinib-miR-34a ratios by combining erlotinib at concentrations from 0.41–100 µM with miR-34a at concentrations from 0.12–30 nM. Drug doses were increased in 2.5-fold increments, and each drug was also used alone as controls. This matrix yielded 49 drug combinations representing 13 different drug ratios (**Fig. 3A**). Levels of cancer cell inhibition, CI and DRI values were determined for each combination and graphed in CI plots, isobolograms and curve-shift diagrams. For the sake of this exercise, we focused on combinations in which miR-34a and erlotinib were added in an IC_50_:IC_50_ ratio (molar ratio 1∶3333) and the following molar-based ratios: 1∶533, 1∶1333, 1∶8333 and 1∶20833.

Calculated CI values predict that erlotinib and miR-34a combined at all of these ratios provide strong synergy (**Fig. 3B**). At effect levels greater than 75% inhibition, CI values were below 0.2. The ratios that contained higher amounts of erlotinib provided lower synergy at effect levels below ∼75% and were slightly superior at effect levels above 75% inhibition. Similarly, the isobologram indicates strong synergy for various erlotinib-miR-34a ratios (**Fig. 3C**). Actual data points demonstrate that 30 nM miR-34a or 100 µM erlotinib are required to induce ∼80% cancer cell inhibition when used as single agents. In contrast, the required dose levels of erlotinib in the combination were substantially decreased as miR-34a amounts were increased. For instance, merely 2.56 µM erlotinib was needed to induce ∼80% inhibition when used with 12 nM miR-34a, thereby reducing the dose requirement of erlotinib by ∼40-fold. Further evidence for the synergistic action of these ratios comes from curve-shift analyses that reveal much lower IC_50_ equivalents of the combination compared with IC_50_ values of the single agents alone (**Fig. 3D**). The IC_50_ eq data correlate with CI data showing dose-dependent degrees of synergy among various ratios: low ratios show lower synergy at low effect levels which is reversed at high levels of cancer cell inhibition.

The full range of 49 combinations was also tested in H1299, H460 and H226 cells and confirmed the results obtained with A549 cells (**Fig. S3**). Multiple ratios provided good synergy, and the ones with higher potency clustered to the ones with higher drug concentrations. Among these were many that met our cut-offs and produced >75% cancer cell inhibition, CI <0.6, and DRI >2 for each drug.

### Erlotinib and miR-34a Cooperate Synergistically in Hepatocellular Carcinoma Cells

**Figure 4:**
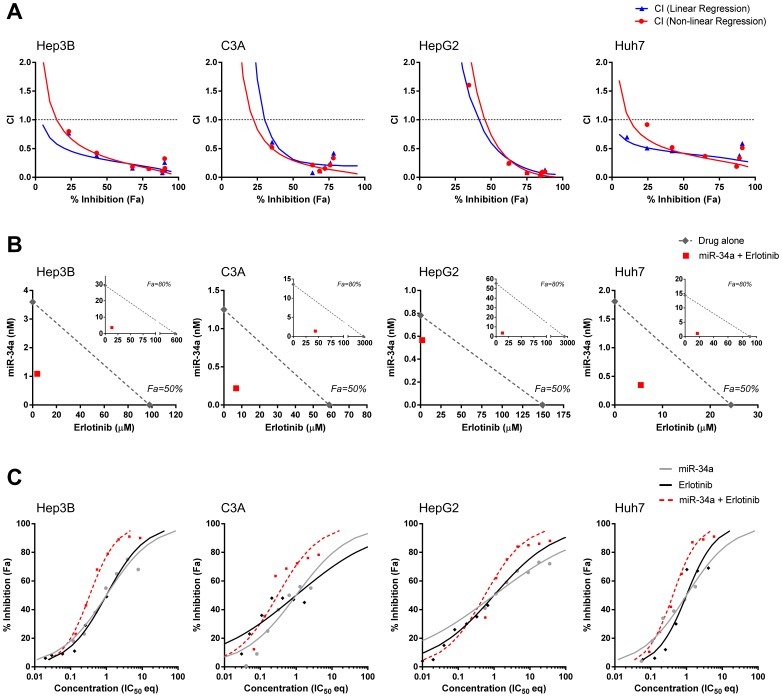
Erlotinib and miR-34a synergize in HCC cells. (**A**) Combination index analysis. (**B**) Isobologram analysis. (**C**) Curve shift analysis. See legend to Figure 2 for explanation of graphs.

To investigate whether the cooperative activity of erlotinib and miR-34a has utility in other cancer indications, we probed this combination in cell models of hepatocellular carcinoma. Liver cancer was chosen as test platform because erlotinib is moderately effective in patients with advanced liver as a single agent and failed to prolong overall survival and time-to-progression in combination with sorafenib [43]–[45]. In addition, MRX34, a miR-34a liposome currently in clinical testing, effectively eliminated liver tumors in preclinical animal studies and therefore may be an attractive agent in combination with erlotinib. Cell models used included Hep3B, C3A, HepG2 and Huh7, several of which showed an upregulation of erlotinib-resistance genes, *AXL*, *HGF*, *FGFR1* and *ERBB3* in comparison to an erlotinib-sensitive lung cancer line (**Fig. S4**). Collectively, levels of miR-34 family members were low or undetectable in liver cancer cells. In agreement with our expectation, IC_50_ values of erlotinib were 25 µM or greater in these four cell lines (**Fig. S5**). The IC_50_ values of miR-34a ranged between 0.3 and 2.3 nM and, thus, were similar to those in lung cancer cells. These values were used as a guide to combine erlotinib and miR-34a at a fixed ratio of IC_50_:IC_50_ and to produce CI, isoboles and IC_50_ eq values (**Fig. 4**). In addition, each combination was also tested in a matrix of different concentrations to assess the combinatorial effects across multiple ratios (**Fig. S6**). Our data predict strong synergy between erlotinib and miR-34a in all cell lines tested. Synergy was observed at high levels of cancer cell inhibition and, hence, occurs within the desirable range of activity (**Fig. 4A**). This result is confirmed by the IC_50_ eq curve shift analyses indicating synergy at higher dose and effect levels. The analysis also shows that the maximal inhibitory activity of the combination is substantially expanded compared to those of the single agents (**Fig. 4C**). Isobolograms demonstrate a stark reduction of the erlotinib dose when used with miR-34a to induce 50% inhibition or greater, such as 80% (**Fig. 4B**). In combination, erlotinib can be used at concentrations as low as 2 µM to inhibit cancer cells by 50%, thereby lowering its dose by 75-fold compared to its single use (see HepG2). Synergy is not limited to a specific ratio but is apparent across most ratios tested (**Fig. S6**). Thus, the data are similar to those generated in lung cancer cells and predict enhanced efficacy for the erlotinib-miR-34a combination in cancers where erlotinib alone is insufficient.

## Discussion

An accurate evaluation of drug-drug interactions is complex because outcomes depend on drug ratios, drug concentrations and desired potency [31]. To investigate the pharmacological relationship between miR-34a mimics and erlotinib, we used multiple analytical approaches to reveal drug enhancements (“Fixed Concentration” model) and to distinguish between additivity, antagonism and synergy (“Fixed Ratio” model). We examined CI values, isobolograms and IC_50_ equivalents derived from linear or non-linear data regression. Our data show that miR-34a augments the sensitivity to erlotinib in all cancer cells tested – whether they were associated with primary or secondary/acquired resistance. A plausible explanation is provided by the fact that tumor suppressor miRNAs inhibit numerous cancer pathways. In support of this hypothesis, *AXL* and *MET*, gene products specifically linked to erlotinib resistance, are directly repressed by miR-34a [27]–[29]. Unexpectedly, erlotinib also enhanced the therapeutic effects of the miR-34a mimic, despite existing evidence implicating miR-34a in the control of multiple oncogenic signaling pathways, including the EGFR pathway [46]. Thus, this result demonstrates that a miRNA mimic can synergize with a single gene-directed therapy and invites the search for other combinations. Based on our data, we speculate that miR-34a may also synergize with other EGFR inhibitors, such as gefitinib, afatinib, panitumumab and cetuximab, as well as HER2 inhibitors such as lapatinib, pertuzumab and trastuzumab.

In lung cancer cells with acquired resistance (HCC827^res^), adding a small amount of miR-34a was capable of reducing erlotinib IC_50_ values below 0.1 µM. This is a remarkable result and suggests that miR-34a can render this cell line equally erlotinib-sensitive compared to parental HCC827 cells. In lung cancer cells with primary resistance, the IC_50_ dose requirement for erlotinib decreased by 4- to 46-fold and was approximately 2 µM. This may be within the range of concentrations that have clinical utility [2]. Erlotinib is given as a daily, oral dose of up to 150 mg. Although the clinical dose level of MRX34 has yet to be established, the molar ratios between miR-34a and erlotinib used in the clinic are likely within the range of ratios that have shown synergy in our cell studies. Further studies are warranted to confirm the superior activity of the combination *in vivo* which will consider administration routes, absorption and pharmacokinetic properties specific to each drug.

Erlotinib is currently used as a first-line therapy for NSCLC patients with activating *EGFR* mutations. It is also used as a maintenance therapy after chemotherapy and second- and third-line therapy for locally advanced or metastatic NSCLC that has failed at least one prior chemotherapy regimen. Clinical trials failed to demonstrate a survival benefit of erlotinib in combination with cisplatin/gemcitabine or carboplatin/paclitaxel compared to conventional chemotherapies alone [2], [47]. A recent Phase III trial, investigating erlotinib plus sorafenib in HCC, also did not meet its endpoint [45]. Thus, other approaches for combination therapies are desired. Our data show that the erlotinib plus miR-34a combination is particularly effective and may substantially broaden the NSCLC patient population that can be treated with erlotinib. The combination was similarly synergistic in HCC cells, suggesting that the synergistic interaction is presumably a result of their molecular mechanisms of action and can also be applied to cancers outside of NSCLC. Since MRX34, a liposomal nanoparticle loaded with synthetic miR-34a mimics, has recently a phase 1 clinical trial [21], clinical testing of the erlotinib-miR-34a combination could be quickly initiated.

## Supporting Information

Figure S1 **Endogenous miR-34 and mRNA levels of genes controlling erlotinib resistance in NSCLC cells.** Total RNA was used in triplicate qRT-PCR to measure miR-34a/b/c and mRNA levels of genes implicated in erlotinib resistance. Data were normalized to house-keeping miRNAs and mRNAs, respectively, and expressed as percent change compared to levels in HCC827 cells. u, undetected.
(TIF)

Figure S2 **Dose-response curves of the single agents in NSCLC cells resistant to erlotinib.** Cells were treated in triplicates with erlotinib or miR-34a alone at indicated concentrations. Cellular proliferation was measured 3 days or 4 days after erlotinib treatment or miR-34a reverse-transfection, respectively. Non-linear regression trendlines were generated using Graphpad, and IC_50_ and IC_25_ values were calculated. Goodness of fit of non-linear regression trendlines is indicated by R^2^ values. The asterisk denotes theoretical IC_50_ values derived from an extrapolation of the dose-response curve (H226).
(TIF)

Figure S3 **Summary table showing potency, CI and DRI values of erlotinib and miR-34a combined at various concentrations and ratios in NSCLC cells.** Combinations that yield Fa >65%, CI <0.6, DRI >2 are highlighted in grey and are considered relevant. Fa, fraction affected (% inhibition of cellular proliferation); CI, combination index; DRI, dose reduction index.
(TIF)

Figure S4 **Endogenous expression of miR-34 and mRNAs of genes controlling erlotinib resistance in HCC cells.** Total RNA was used in triplicate qRT-PCR to measure miR-34a/b/c and mRNA levels of genes implicated in erlotinib resistance. Data were normalized to house-keeping miRNAs and mRNAs, respectively, and expressed as percent change compared to levels in HCC827 cells. u, undetected.
(TIF)

Figure S5 **Dose-response curves of the single agents in HCC cells resistant to erlotinib.** Cells were treated in triplicates with erlotinib or miR-34a alone at indicated concentrations. Cellular proliferation was measured 3 days or 6 days after erlotinib treatment or miR-34a reverse-transfection, respectively. Non-linear regression trendlines were generated using Graphpad, and IC_50_ and IC_25_ values were calculated. Goodness of fit of non-linear regression trendlines is indicated by R^2^ values. The asterisk denotes theoretical IC_50_ values of erlotinib derived from an extrapolation of the dose-response curve (Hep3B, C3A, HepG2).
(TIF)

Figure S6 **Summary table showing potency, CI and DRI values of erlotinib and miR-34a combined at various concentrations and ratios in HCC cells.** Combinations that yield Fa >65%, CI <0.6, DRI >2 are highlighted in grey and are considered relevant. Fa, fraction affected (% inhibition of cellular proliferation); CI, combination index; DRI, dose reduction index.
(TIF)

## References

[1] (2013) American Cancer Society. Cancer Facts & Figures 2013. Available: www.cancer.org.

[2] Sharma SV, Bell DW, Settleman J Haber DA (2007) Epidermal growth factor receptor mutations in lung cancer. Nat Rev Cancer 7: 169–81. 17318210 10.1038/nrc2088

[3] Bell DW, Lynch TJ, Haserlat SM, Harris PL, Okimoto RA, et al (2005) Epidermal growth factor receptor mutations and gene amplification in non-small-cell lung cancer: molecular analysis of the IDEAL/INTACT gefitinib trials. J Clin Oncol 23: 8081–92. 16204011 10.1200/JCO.2005.02.7078

[4] Fukuoka M, Yano S, Giaccone G, Tamura T, Nakagawa K, et al (2003) Multi-institutional randomized phase II trial of gefitinib for previously treated patients with advanced non-small-cell lung cancer (The IDEAL 1 Trial) [corrected]. J Clin Oncol 21: 2237–46. 12748244 10.1200/JCO.2003.10.038

[5] Gazdar AF, Shigematsu H, Herz J, Minna JD (2004) Mutations and addiction to EGFR: the Achilles 'heal' of lung cancers? Trends Mol Med 10: 481–6. 15464447 10.1016/j.molmed.2004.08.008

[6] Pao W, Wang TY, Riely GJ, Miller VA, Pan Q, et al (2005) KRAS mutations and primary resistance of lung adenocarcinomas to gefitinib or erlotinib. PLoS Med 2: e17. 15696205 10.1371/journal.pmed.0020017 PMC545207

[7] Pao W, Miller VA, Politi KA, Riely GJ, Somwar R, et al (2005) Acquired resistance of lung adenocarcinomas to gefitinib or erlotinib is associated with a second mutation in the EGFR kinase domain. PLoS Med 2: e73. 15737014 10.1371/journal.pmed.0020073 PMC549606

[8] Engelman JA, Zejnullahu K, Mitsudomi T, Song Y, Hyland C, et al (2007) MET amplification leads to gefitinib resistance in lung cancer by activating ERBB3 signaling. Science 316: 1039–43. 17463250 10.1126/science.1141478

[9] Byers LA, Diao L, Wang J, Saintigny P, Girard L, et al (2012) An epithelial-mesenchymal transition gene signature predicts resistance to EGFR and PI3K inhibitors and identifies Axl as a therapeutic target for overcoming EGFR inhibitor resistance. Clin Cancer Res 19: 279–90. 23091115 10.1158/1078-0432.CCR-12-1558 PMC3567921

[10] Zhang Z, Lee JC, Lin L, Olivas V, Au V, et al (2012) Activation of the AXL kinase causes resistance to EGFR-targeted therapy in lung cancer. Nat Genet 44: 852–60. 22751098 10.1038/ng.2330 PMC3408577

[11] Engelman JA, Mukohara T, Zejnullahu K, Lifshits E, Borras AM, et al (2006) Allelic dilution obscures detection of a biologically significant resistance mutation in EGFR-amplified lung cancer. J Clin Invest 116: 2695–706. 16906227 10.1172/JCI28656 PMC1570180

[12] Kawano O, Sasaki H, Endo K, Suzuki E, Haneda H, et al (2006) PIK3CA mutation status in Japanese lung cancer patients. Lung Cancer 54: 209–15. 16930767 10.1016/j.lungcan.2006.07.006

[13] Sos ML, Koker M, Weir BA, Heynck S, Rabinovsky R, et al (2009) PTEN loss contributes to erlotinib resistance in EGFR-mutant lung cancer by activation of Akt and EGFR. Cancer Res 69: 3256–61. 19351834 10.1158/0008-5472.CAN-08-4055 PMC2849653

[14] Gong Y, Yao E, Shen R, Goel A, Arcila M, et al (2009) High expression levels of total IGF-1R and sensitivity of NSCLC cells in vitro to an anti-IGF-1R antibody (R1507). PLoS One 4: e7273. 19806209 10.1371/journal.pone.0007273 PMC2752171

[15] Sharma SV, Lee DY, Li B, Quinlan MP, Takahashi F, et al (2010) A chromatin-mediated reversible drug-tolerant state in cancer cell subpopulations. Cell 141: 69–80. 20371346 10.1016/j.cell.2010.02.027 PMC2851638

[16] Bader AG, Brown D, Winkler M (2010) The promise of microRNA replacement therapy. Cancer Res 70: 7027–30. 20807816 10.1158/0008-5472.CAN-10-2010 PMC2940943

[17] Ling H, Fabbri M, Calin GA (2013) MicroRNAs and other non-coding RNAs as targets for anticancer drug development. Nat Rev Drug Discov 12: 847–65. 24172333 10.1038/nrd4140 PMC4548803

[18] Ji Q, Hao X, Meng Y, Zhang M, Desano J, et al (2008) Restoration of tumor suppressor miR-34 inhibits human p53-mutant gastric cancer tumorspheres. BMC Cancer 8: 266. 18803879 10.1186/1471-2407-8-266 PMC2564978

[19] Liu C, Kelnar K, Liu B, Chen X, Calhoun-Davis T, et al (2011) The microRNA miR-34a inhibits prostate cancer stem cells and metastasis by directly repressing CD44. Nat Med 17: 211–5. 21240262 10.1038/nm.2284 PMC3076220

[20] Bader AG (2012) miR-34 - a microRNA replacement therapy is headed to the clinic. Front Genet 3: 120. 22783274 10.3389/fgene.2012.00120 PMC3387671

[21] (2013) Mirna Therapeutics. Press Release: Mirna Therapeutics is First to Advance MicroRNA into the Clinic for Cancer. Corporate website.

[22] Akao Y, Noguchi S, Iio A, Kojima K, Takagi T, et al (2010) Dysregulation of microRNA-34a expression causes drug-resistance to 5-FU in human colon cancer DLD-1 cells. Cancer Lett 300: 197–204. 21067862 10.1016/j.canlet.2010.10.006

[23] Fujita Y, Kojima K, Hamada N, Ohhashi R, Akao Y, et al (2008) Effects of miR-34a on cell growth and chemoresistance in prostate cancer PC3 cells. Biochem Biophys Res Commun 377: 114–9. 18834855 10.1016/j.bbrc.2008.09.086

[24] Kojima K, Fujita Y, Nozawa Y, Deguchi T, Ito M (2010) MiR-34a attenuates paclitaxel-resistance of hormone-refractory prostate cancer PC3 cells through direct and indirect mechanisms. Prostate 70: 1501–12. 20687223 10.1002/pros.21185

[25] Vinall RL, Ripoll AZ, Wang S, Pan CX, deVere White RW (2011) MiR-34a chemosensitizes bladder cancer cells to cisplatin treatment regardless of p53-Rb pathway status. Int J Cancer 130: 2526–38. 21702042 10.1002/ijc.26256 PMC4568996

[26] Weeraratne SD, Amani V, Neiss A, Teider N, Scott DK, et al (2010) miR-34a confers chemosensitivity through modulation of MAGE-A and p53 in medulloblastoma. Neuro Oncol 13: 165–75. 21177782 10.1093/neuonc/noq179 PMC3064629

[27] Kaller M, Liffers ST, Oeljeklaus S, Kuhlmann K, Roh S, et al.. (2011) Genome-wide characterization of miR-34a induced changes in protein and mRNA expression by a combined pulsed SILAC and microarray analysis. Mol Cell Proteomics 10: M111 010462. 10.1074/mcp.M111.010462 PMC3149097 21566225

[28] Mudduluru G, Ceppi P, Kumarswamy R, Scagliotti GV, Papotti M, et al (2011) Regulation of Axl receptor tyrosine kinase expression by miR-34a and miR-199a/b in solid cancer. Oncogene 30: 2888–99. 21317930 10.1038/onc.2011.13

[29] He L, He X, Lim LP, de Stanchina E, Xuan Z, et al (2007) A microRNA component of the p53 tumour suppressor network. Nature 447: 1130–4. 17554337 10.1038/nature05939 PMC4590999

[30] Zhao J, Lammers P, Torrance CJ, Bader AG (2013) TP53-independent function of miR-34a via HDAC1 and p21(CIP1/WAF1.). Mol Ther 21: 1678–86. 23836017 10.1038/mt.2013.148 PMC3776635

[31] Chou TC (2010) Drug combination studies and their synergy quantification using the Chou-Talalay method. Cancer Res 70: 440–6. 20068163 10.1158/0008-5472.CAN-09-1947

[32] Tallarida RJ (2001) Drug synergism: its detection and applications. J Pharmacol Exp Ther 298: 865–72. 11504778

[33] Tallarida RJ (2006) An overview of drug combination analysis with isobolograms. J Pharmacol Exp Ther 319: 1–7. 16670349 10.1124/jpet.106.104117

[34] ComboSyn, Inc. website. Available: http://www.combosyn.com. Accessed 2014 Jan 23.

[35] Zhao L, Au JL, Wientjes MG (2010) Comparison of methods for evaluating drug-drug interaction. Front Biosci (Elite Ed) 2: 241–9. 20036874 10.2741/e86 PMC2885905

[36] Giles KM, Kalinowski FC, Candy PA, Epis MR, Zhang PM, et al.. (2013) Axl mediates acquired resistance of head and neck cancer cells to the epidermal growth factor receptor inhibitor erlotinib. Mol Cancer Ther. 10.1158/1535-7163.MCT-13-0170 24026012

[37] COSMIC website (Catalogue of Somatic Mutations in Cancer). Available: http://cancer.sanger.ac.uk/cancergenome/projects/cosmic/. Accessed 2014 Jan 23.

[38] Fujimoto N, Wislez M, Zhang J, Iwanaga K, Dackor J, et al (2005) High expression of ErbB family members and their ligands in lung adenocarcinomas that are sensitive to inhibition of epidermal growth factor receptor. Cancer Res 65: 11478–85. 16357156 10.1158/0008-5472.CAN-05-1977

[39] Witta SE, Gemmill RM, Hirsch FR, Coldren CD, Hedman K, et al (2006) Restoring E-cadherin expression increases sensitivity to epidermal growth factor receptor inhibitors in lung cancer cell lines. Cancer Res 66: 944–50. 16424029 10.1158/0008-5472.CAN-05-1988

[40] Janmaat ML, Kruyt FA, Rodriguez JA Giaccone G (2003) Response to epidermal growth factor receptor inhibitors in non-small cell lung cancer cells: limited antiproliferative effects and absence of apoptosis associated with persistent activity of extracellular signal-regulated kinase or Akt kinase pathways. Clin Cancer Res 9: 2316–26. 12796401

[41] Ono M, Hirata A, Kometani T, Miyagawa M, Ueda S, et al (2004) Sensitivity to gefitinib (Iressa, ZD1839) in non-small cell lung cancer cell lines correlates with dependence on the epidermal growth factor (EGF) receptor/extracellular signal-regulated kinase 1/2 and EGF receptor/Akt pathway for proliferation. Mol Cancer Ther 3: 465–72. 15078990

[42] Van Schaeybroeck S, Kyula J, Kelly DM, Karaiskou-McCaul A, Stokesberry SA, et al (2006) Chemotherapy-induced epidermal growth factor receptor activation determines response to combined gefitinib/chemotherapy treatment in non-small cell lung cancer cells. Mol Cancer Ther 5: 1154–65. 16731747 10.1158/1535-7163.MCT-05-0446

[43] Philip PA, Mahoney MR, Allmer C, Thomas J, Pitot HC, et al (2005) Phase II study of Erlotinib (OSI-774) in patients with advanced hepatocellular cancer. J Clin Oncol 23: 6657–63. 16170173 10.1200/JCO.2005.14.696

[44] Thomas MB, Chadha R, Glover K, Wang X, Morris J, et al (2007) Phase 2 study of erlotinib in patients with unresectable hepatocellular carcinoma. Cancer 110: 1059–67. 17623837 10.1002/cncr.22886

[45] Zhu AX, Rosmorduc O, Evans J, Ross P, Santoro A, et al.. (2012) SEARCH: A phase III, randomized, double-blind, placebo-controlled trial of sorafenib plus erlotinib in patients with hepatocellular carcinoma (HCC). 37th Annual European Society for Medical Oncology Congress, Vienna, Austria, September 28-October 2 (abstr 917).

[46] Lal A, Thomas MP, Altschuler G, Navarro F, O'Day E, et al (2011) Capture of microRNA-bound mRNAs identifies the tumor suppressor miR-34a as a regulator of growth factor signaling. PLoS Genet 7: e1002363. 22102825 10.1371/journal.pgen.1002363 PMC3213160

[47] Herbst RS, Prager D, Hermann R, Fehrenbacher L, Johnson BE, et al (2005) TRIBUTE: a phase III trial of erlotinib hydrochloride (OSI-774) combined with carboplatin and paclitaxel chemotherapy in advanced non-small-cell lung cancer. J Clin Oncol 23: 5892–9. 16043829 10.1200/JCO.2005.02.840

