# Supplementary figures and images for: In-Depth Analysis Shows Synergy between Erlotinib and miR-34a
Source: PLoS One. 2014 Feb 14;9(2):e89105. doi: 10.1371/journal.pone.0089105 (PMC3925231; doi:10.1371/journal.pone.0089105)

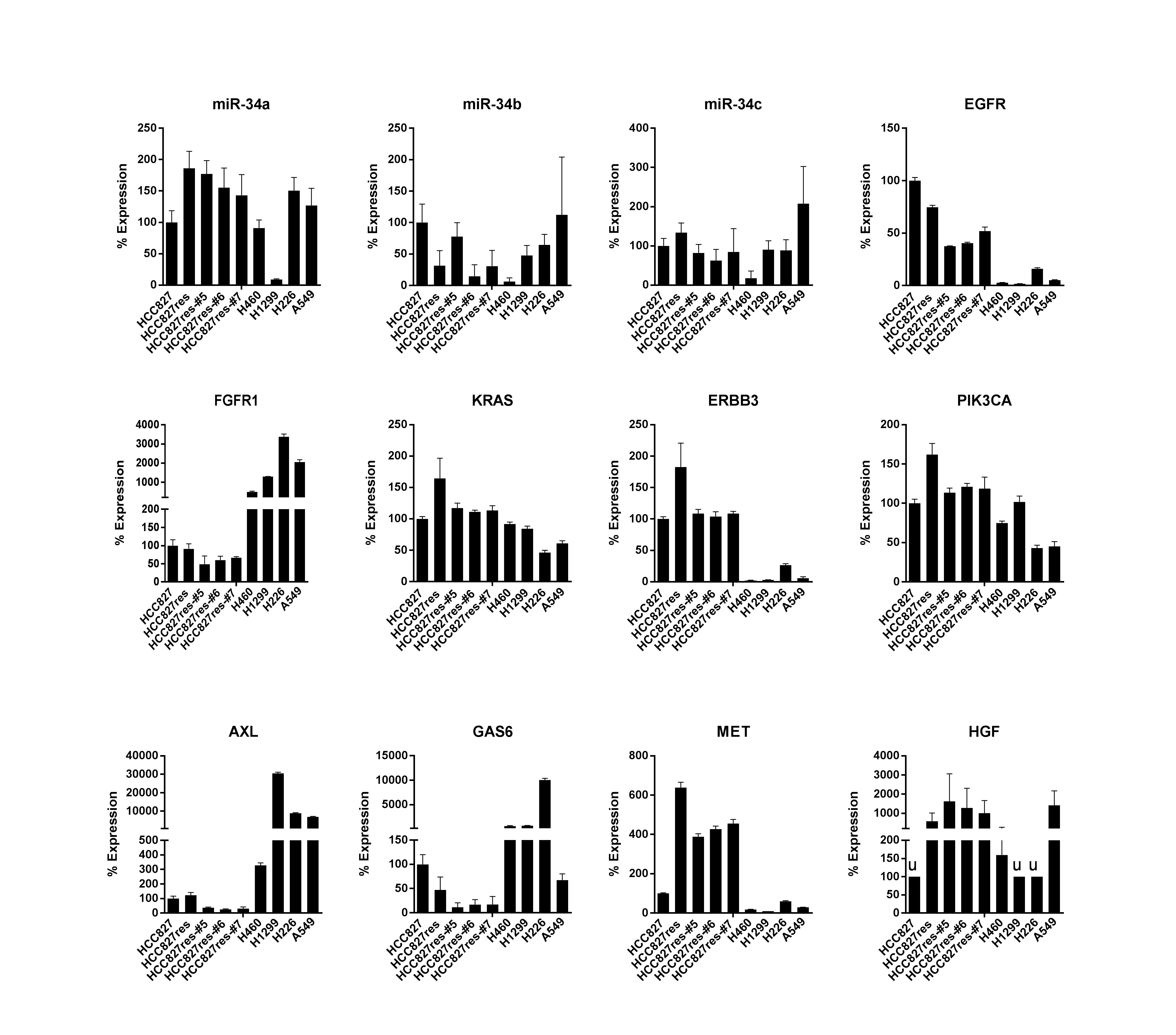

Supplement: Figure S1 — Endogenous miR-34 and mRNA levels of genes controlling erlotinib resistance in NSCLC cells. Total RNA was used in triplicate qRT-PCR to measure miR-34a/b/c and mRNA levels of genes implicated in erlotinib resistance. Data were normalized to house-keeping miRNAs and mRNAs, respectively, and expressed as percent change compared to levels in HCC827 cells. u, undetected. (TIF) [file pone.0089105.s001.tif]

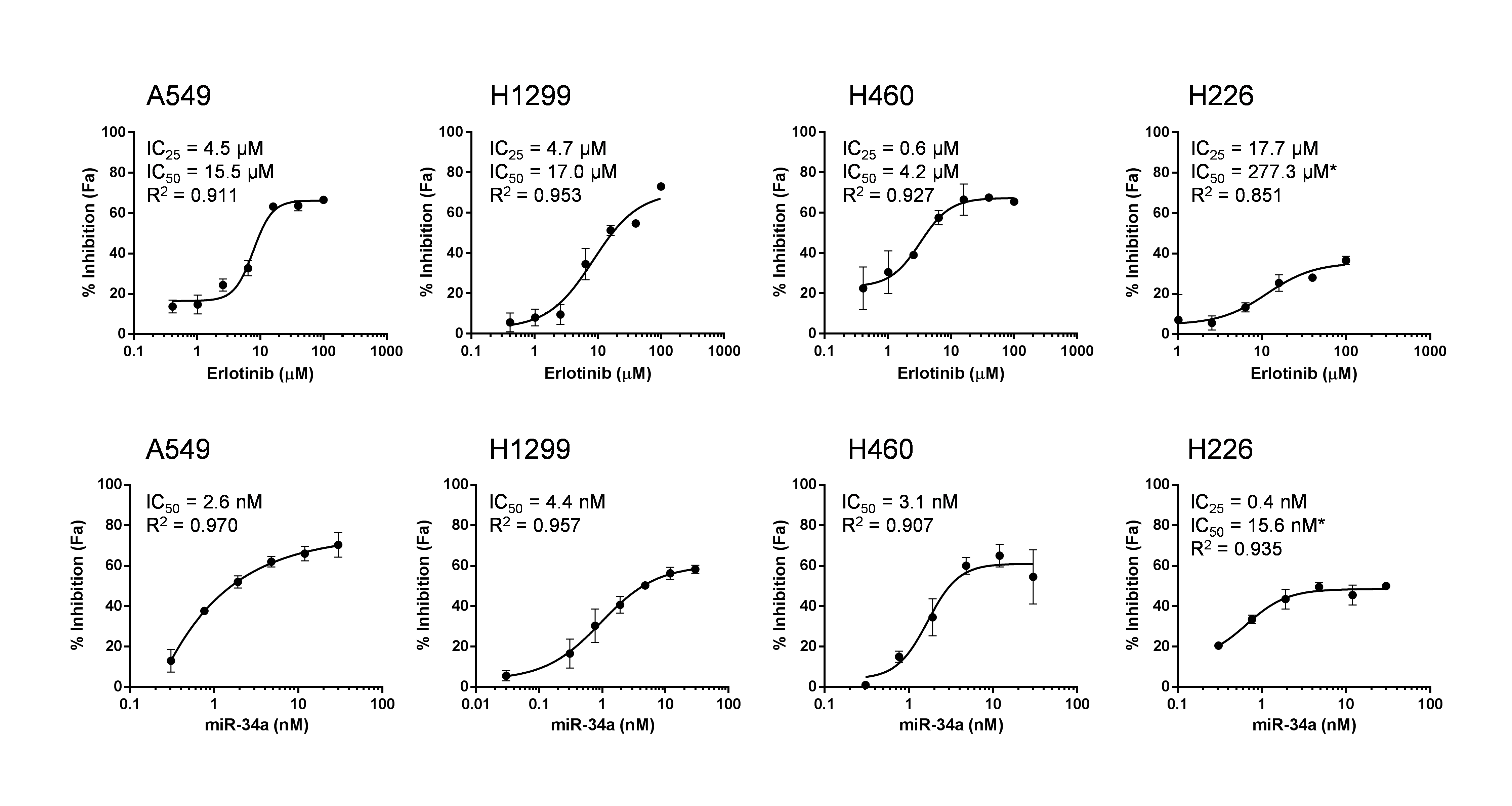

Supplement: Figure S2 — Dose-response curves of the single agents in NSCLC cells resistant to erlotinib. Cells were treated in triplicates with erlotinib or miR-34a alone at indicated concentrations. Cellular proliferation was measured 3 days or 4 days after erlotinib treatment or miR-34a reverse-transfection, respectively. Non-linear regression trendlines were generated using Graphpad, and IC50 and IC25 values were calculated. Goodness of fit of non-linear regression trendlines is indicated by R2 values. The asterisk denotes theoretical IC50 values derived from an extrapolation of the dose-response curve (H226). (TIF) [file pone.0089105.s002.tif]

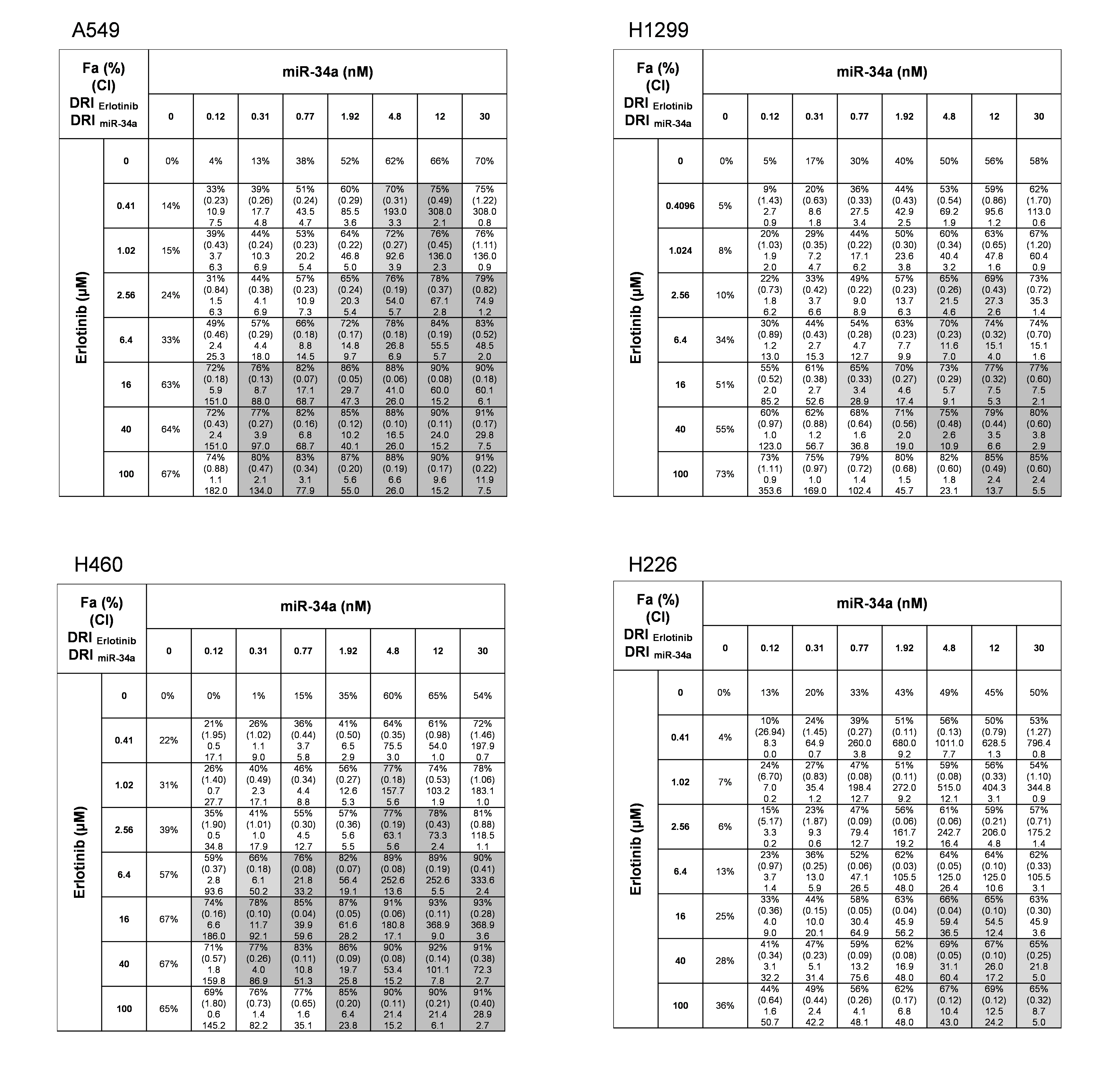

Supplement: Figure S3 — Summary table showing potency, CI and DRI values of erlotinib and miR-34a combined at various concentrations and ratios in NSCLC cells. Combinations that yield Fa >65%, CI <0.6, DRI >2 are highlighted in grey and are considered relevant. Fa, fraction affected (% inhibition of cellular proliferation); CI, combination index; DRI, dose reduction index. (TIF) [file pone.0089105.s003.tif]

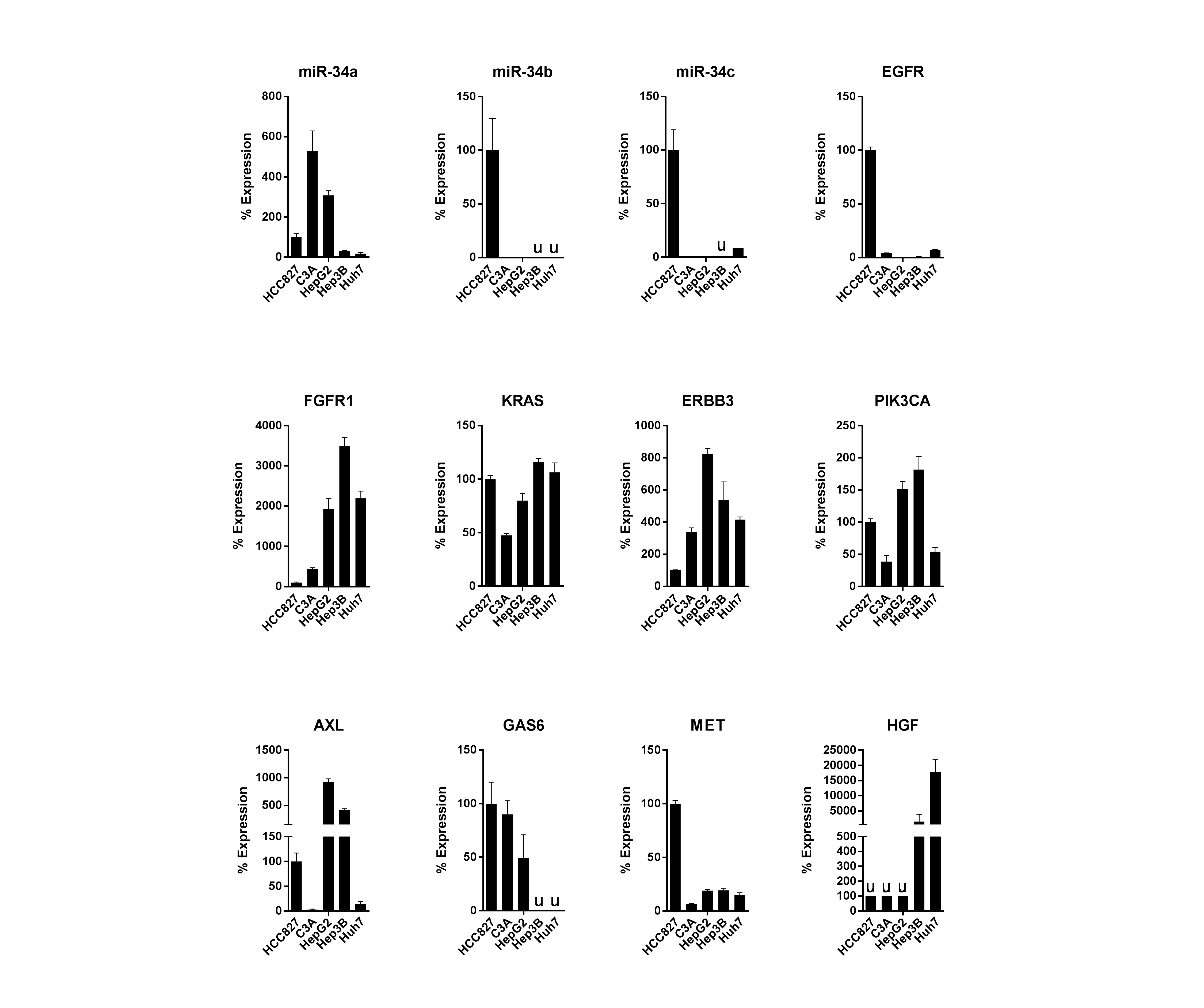

Supplement: Figure S4 — Endogenous expression of miR-34 and mRNAs of genes controlling erlotinib resistance in HCC cells. Total RNA was used in triplicate qRT-PCR to measure miR-34a/b/c and mRNA levels of genes implicated in erlotinib resistance. Data were normalized to house-keeping miRNAs and mRNAs, respectively, and expressed as percent change compared to levels in HCC827 cells. u, undetected. (TIF) [file pone.0089105.s004.tif]

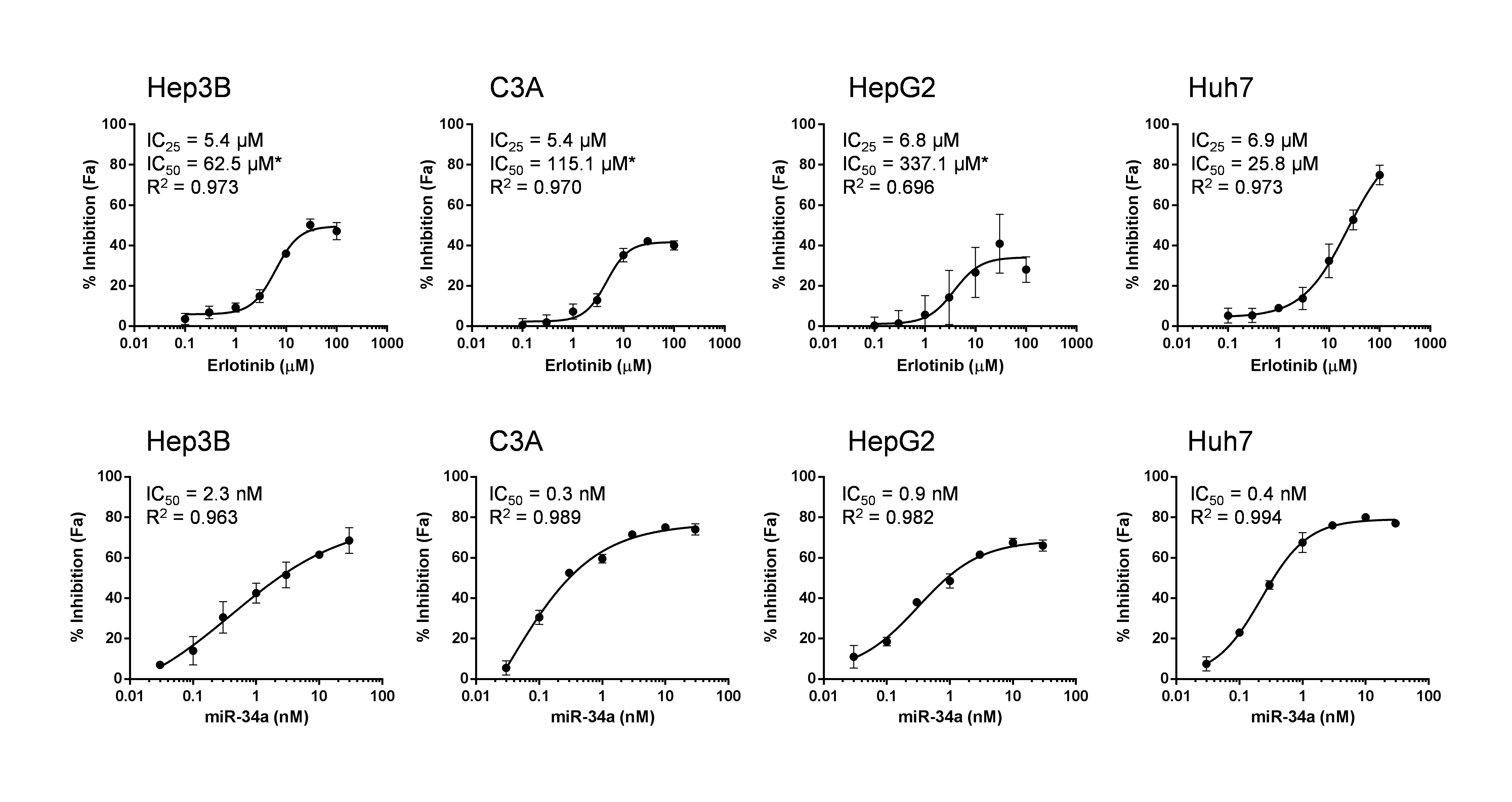

Supplement: Figure S5 — Dose-response curves of the single agents in HCC cells resistant to erlotinib. Cells were treated in triplicates with erlotinib or miR-34a alone at indicated concentrations. Cellular proliferation was measured 3 days or 6 days after erlotinib treatment or miR-34a reverse-transfection, respectively. Non-linear regression trendlines were generated using Graphpad, and IC50 and IC25 values were calculated. Goodness of fit of non-linear regression trendlines is indicated by R2 values. The asterisk denotes theoretical IC50 values of erlotinib derived from an extrapolation of the dose-response curve (Hep3B, C3A, HepG2). (TIF) [file pone.0089105.s005.tif]

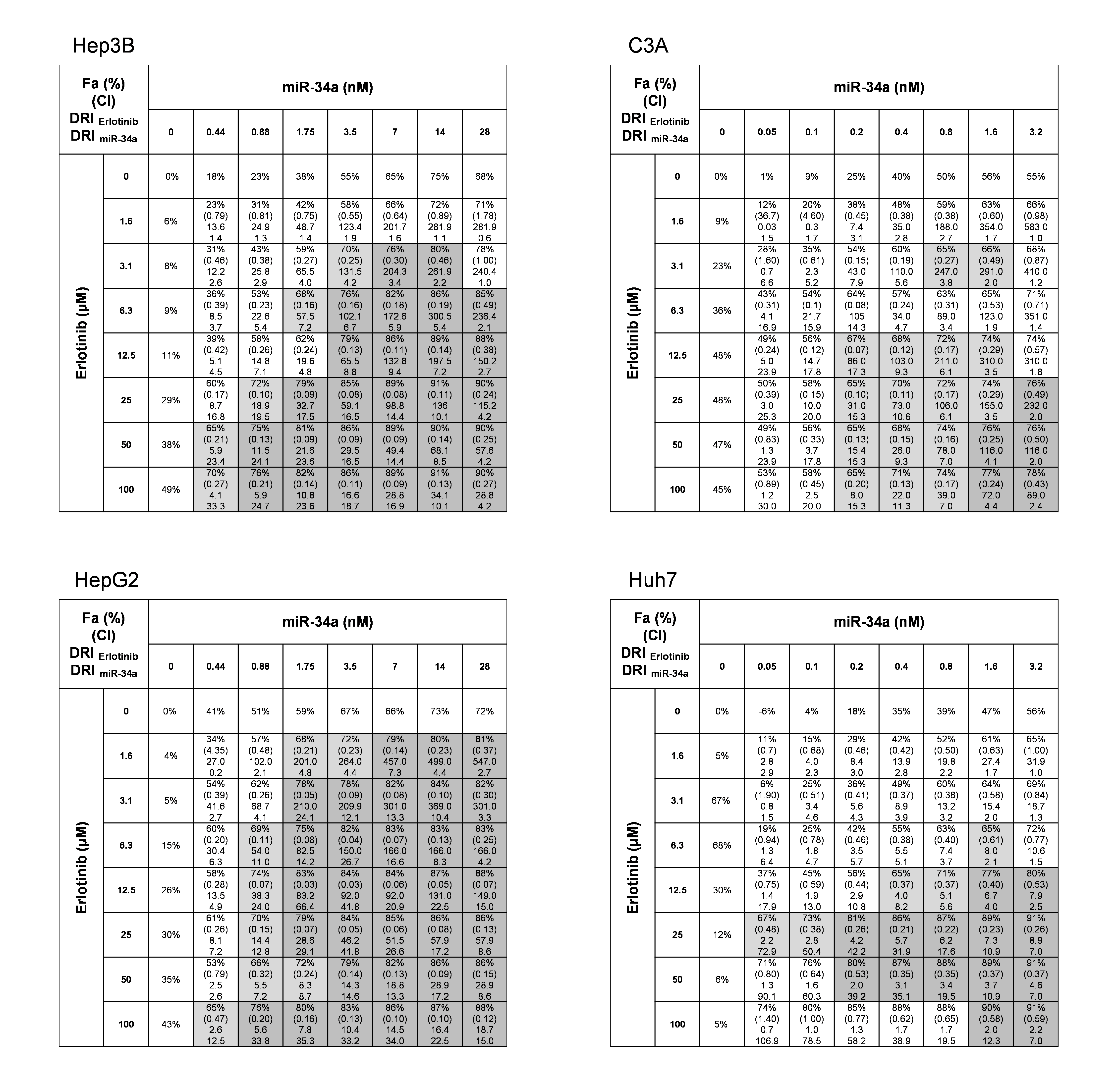

Supplement: Figure S6 — Summary table showing potency, CI and DRI values of erlotinib and miR-34a combined at various concentrations and ratios in HCC cells. Combinations that yield Fa >65%, CI <0.6, DRI >2 are highlighted in grey and are considered relevant. Fa, fraction affected (% inhibition of cellular proliferation); CI, combination index; DRI, dose reduction index. (TIF) [file pone.0089105.s006.tif]
